# Supplementary material for: OES-Fed: a federated learning framework in vehicular network based on noise data filtering
Source: PeerJ Comput Sci. 2022 Sep 20;8:e1101. doi: 10.7717/peerj-cs.1101 (PMC9575870; doi:10.7717/peerj-cs.1101)
Supplement: Supplemental Information 1 [file peerj-cs-08-1101-s001.pdf]

our-mnist

December 28,2021

[1]:
```
import torch
import torch.nn as nn
import torch.nn.functional as F
import torch.optim as optim
from torchvision import datasets, transforms
from torch.utils.data import DataLoader, Dataset
import syft as sy
import copy
import numpy as np
import time
import random
import warnings

import importlib
importlib.import_module('FLDataset')
from statsmodels.tsa.holtwinters import ExponentialSmoothing,␣
 ↪SimpleExpSmoothing, Holt
from sklearn import preprocessing
from FLDataset import load_dataset, getActualImgs, load_dataset_cifar
from utils import averageModels
```

[2]:
```
# !pip install statsmodels
```

[3]:
```
class Arguments():
    def __init__(self):
        self.images = 60000
        self.clients = 40
        self.rounds = 30
        self.epochs = 10
        self.local_batches = 64
        self.lr = 0.01
        self.C = 0.9
        self.drop_rate = 0.1
        self.torch_seed = 0
        self.log_interval = 10
        self.iid = 'non-iid'
        self.split_size = int(self.images / self.clients)
```

```
        self.samples = self.split_size / self.images
        self.use_cuda = False
        self.save_model = False
        self.step = 2
        self.count = 0

args = Arguments()

use_cuda = args.use_cuda and torch.cuda.is_available()
device = torch.device("cuda" if use_cuda else "cpu")
kwargs = {'num_workers': 1, 'pin_memory': True} if use_cuda else {}
```

[4]:
```
hook = sy.TorchHook(torch)
clients = []

for i in range(args.clients):
    clients.append({'hook': sy.VirtualWorker(hook, id="client{}".format(i+1))})
```

[5]:
```
global_train, global_test, train_group, test_group = load_dataset(args.clients,␣
 ↪args.iid)
```

```
C:\ProgramData\Anaconda3\lib\site-packages\torchvision\datasets\mnist.py:45:
UserWarning: train_labels has been renamed targets
  warnings.warn("train_labels has been renamed targets")
```

[6]:
```
for inx, client in enumerate(clients):
    trainset_ind_list = list(train_group[inx])
    client['trainset'] = getActualImgs(global_train, trainset_ind_list, args.
 ↪local_batches)
    client['testset'] = getActualImgs(global_test, list(test_group[inx]), args.
 ↪local_batches)
    client['samples'] = len(trainset_ind_list) / args.images
```

[7]:
```
transform = transforms.Compose([transforms.ToTensor(), transforms.Normalize((0.
 ↪1307,), (0.3081,))])
global_test_dataset = datasets.MNIST('./', train=False, download=True,␣
 ↪transform=transform)
global_test_loader = DataLoader(global_test_dataset, batch_size=args.
 ↪local_batches, shuffle=True)
```

[8]:
```
class Net(nn.Module):
    def __init__(self):
        super(Net, self).__init__()
        self.conv1 = nn.Conv2d(1, 20, 5, 1)
        self.conv2 = nn.Conv2d(20, 50, 5, 1)
        self.fc1 = nn.Linear(4*4*50, 500)
        self.fc2 = nn.Linear(500, 10)
```

```
    def forward(self, x):
        x = F.relu(self.conv1(x))
        x = F.max_pool2d(x, 2, 2)
        x = F.relu(self.conv2(x))
        x = F.max_pool2d(x, 2, 2)
        x = x.view(-1, 4*4*50)
        x = F.relu(self.fc1(x))
        x = self.fc2(x)

        return F.log_softmax(x, dim=1)
```

[9]:
```
def ClientUpdate(args, device, client):
    client['model'].train()
    client['model'].send(client['hook'])
    epochs = args.epochs + 1+ lc[y].item()
    epochs = int(epochs)
    for epoch in range(1, epochs):
        for batch_idx, (data, target) in enumerate(client['trainset']):
            data = data.send(client['hook'])
            target = target.send(client['hook'])

            data, target = data.to(device), target.to(device)
            client['optim'].zero_grad()
            output = client['model'](data)
            loss = F.nll_loss(output, target)
            loss.backward()
            client['optim'].step()

#             if batch_idx % args.log_interval == 0:
#                 loss = loss.get()
#                 print('Model {} Train Epoch: {} [{}/{} ({:.0f}%)]\tLoss: {:.
 ↪6f}'.format(
#                     client['hook'].id,
#                     epoch, batch_idx * args.local_batches,␣
 ↪len(client['trainset']) * args.local_batches,
#                     100. * batch_idx / len(client['trainset']), loss))

    client['model'].get()
```

[10]:
```
def test(args, model, device, test_loader, name, rounds):
    model.eval()
    test_loss = 0
    correct = 0
    accuracy = 0
    with torch.no_grad():
        for data, target in test_loader:
```

```
            data, target = data.to(device), target.to(device)
            output = model(data)
            test_loss += F.nll_loss(output, target, reduction='sum').item() #␣
↪sum up batch loss
            pred = output.argmax(1, keepdim=True) # get the index of the max␣
↪log-probability
            correct += pred.eq(target.view_as(pred)).sum().item()

    test_loss /= len(test_loader.dataset)
    accuracy = 100. *correct / len(test_loader.dataset)

#     print('\nTest set{}: Average loss for {} model: {:.4f}\n'.format(rounds,
#         name, test_loss))
    return accuracy,test_loss
```

[11]:
```
def averageModels1(global_model, clients):
    client_models = [clients[i]['model'] for i in range(len(clients))]
    samples = [clients[i]['samples'] for i in range(len(clients))]
    global_dict = global_model.state_dict()
    for k in global_dict.keys():
        global_dict[k] = torch.stack([client_models[i].state_dict()[k].float()␣
↪* count2 for i in range(len(client_models))], 0).sum(0)

    global_model.load_state_dict(global_dict)
    return global_model
```

[12]:
```
def detect_outliers(data1,data2):
    threshold=10
    mean_d = np.mean(data1)
    std_d = np.std(data1)
    outliers = ()

    for y in data2:
        z_score= (y - mean_d)/std_d
        if np.abs(z_score) > threshold:
            outliers = outliers + (y,)
    return outliers
```

[13]:
```
warnings.filterwarnings("ignore")
torch.manual_seed(args.torch_seed)
global_model = Net()
global_model1 = Net()
x = torch.full([args.clients], 0)
lc = torch.full([args.clients], 0)
# step = args.step
acc_list = torch.full([args.rounds,args.clients], 0)
gl_list = torch.full([args.rounds], 0)
```

```
l_list = torch.full([args.rounds], 0)
lqd_list = torch.full([args.rounds], 0)
lb_list = torch.full([args.rounds], 0)
loss_list = torch.full([args.rounds], 0)

for client in clients:
    torch.manual_seed(args.torch_seed)
    client['model'] = Net().to(device)
    client['optim'] = optim.SGD(client['model'].parameters(), lr=args.lr)

for fed_round in range(args.rounds):

    # number of selected clients
    m = int(max(args.C * args.clients, 1))

    # Selected devices
    np.random.seed(fed_round)
    selected_clients_inds = np.random.choice(range(len(clients)), m,␣
↪replace=False)
    selected_clients = [clients[i] for i in selected_clients_inds]

    # Active devices
    np.random.seed(fed_round)
    active_clients_inds = np.random.choice(selected_clients_inds, int((1-args.
↪drop_rate) * m), replace=False)
    active_clients = [clients[i] for i in active_clients_inds]
    fail_inds = ()
    for s in range(0,args.clients):
        n = 0
        for d in range(len(active_clients_inds)):
            if(active_clients_inds[d]==s):
                n += 1
        if(n<1):
            fail_inds = fail_inds+(s,)

    # Training
    up = 0
    for client in active_clients:
        y = active_clients_inds[up].item()
        ClientUpdate(args, device, client)
        x[y] = x[y]+args.epochs+lc[y]
        up += 1


    Acc = ()
    u = 0
    for client in active_clients:
```

```
        accuracy,test_loss = test(args, client['model'], device,␣
↪client['testset'], client['hook'].id, fed_round)
        t = active_clients_inds[u].item()
        acc_list[fed_round][t] = accuracy
        Acc = Acc +(accuracy,)
        u += 1

#     #
#     if(fed_round>=3):
#         acc = ()
#         for xz in range(len(Acc)):
#             shuju = np.zeros(fed_round+1)
#             t = active_clients_inds[xz].item()
#             for xc in range(fed_round+1):
#                 shuju[xc] = acc_list[xc][t]
#             fit3 = SimpleExpSmoothing(shuju).fit(smoothing_level=0.
↪6,optimized=False)
#             fit4 = fit3.forecast(1).item()
#             acc = acc +(fit4,)
# #         print(acc)

    # Averaging
    global_model = averageModels(global_model, active_clients)
    # Testing the average model
    gl_acc,gl_loss =  test(args, global_model, device, global_test_loader,␣
↪'Global', fed_round)
    #
    a1 = sorted(Acc)
    count1 = 0
    for a2 in range(len(a1)):
        if(a1[a2]>=gl_acc):
            count1 +=1
    if(count1 <10):
        count1 = 10

    if(count1>=16):
        if(fed_round+1 ==args.rounds):
            print(x)

        print('\nRound{} set: Average loss for Global model:  Accuracy: ({:.
↪1f}%)\n'.format(fed_round+1,gl_acc))
        for client in clients:
            client['model'].load_state_dict(global_model.state_dict())
    else:
        count2 = (1/(count1*2))
        acc1 =()
        for a3 in range((len(a1)-count1*2),len(a1)):
```

```
        acc1 = acc1+(a1[a3],)
    acc2 = list(acc1)
    Acc1 = list(Acc)

    for a4 in range(len(acc2)):
        for a5 in range(len(Acc)):
            if (acc2[a4]==Acc1[a5]):
                acc2[a4] = a5
                Acc1[a5] = 0
                break

    acc3 = ()
    for a6 in range(len(acc1)):
        l = active_clients_inds[acc2[a6]]
        acc3 = acc3+(l,)

    ac_b = ()
    for a2 in range(len(Acc)):
        if(Acc[a2]>=gl_acc):
            ac_b = ac_b+(Acc[a2],)


    sub_z=()
    z_z = ()
    ac_in = ()
    for ac in ac_b:
        for i in range(len(Acc)):
            if(ac==Acc[i]):
                sub_z = sub_z+(Acc[i],)
                ac_in = ac_in+(i,)
            else:
                z_z = z_z+(Acc[i],)

    sz = ()
    z = ()
    aci = ()
    for u in sub_z:
        if u not in sz:
            sz = sz+(u,)
    for d in z_z:
        if d not in z:
            z = z+(d,)
    for ii in ac_in:
        if ii not in aci:
            aci = aci+(ii,)
    #
    lqd = detect_outliers(sz,z)
```

```
        if lqd == torch.Size([]):
            lqd_list[fed_round] = 0
        else:
            lqd_list[fed_round] = len(lqd)
        #
        i_d = ()
        inds = ()
        for a in lqd:
            for i in range(len(Acc)):
                if(a==Acc[i]):
                    i_d = i_d+(i,)
        for d in i_d:
            if d not in inds:
                inds = inds+(d,)
        for s in inds:
            l = active_clients_inds[s]
            lc[l] = -2
        for i1 in aci:
            l = active_clients_inds[i1]
            lc[l] = 2

        #
        if(fed_round>=3):
            lb  = random.randint(0,4)
            Z = np.mat([i for i in range(500)])
            X = np.mat([[0,], [0,]])
            P = np.mat([[1, 0], [0, 1]])
            F = np.mat([[1, 1], [0, 1]])
            Q = np.mat([[0.0001, 0], [0, 0.0001]])
            H = np.mat([1, 0])
            R = np.mat([1])
            for i in range(100):
                x_predict = F * X
                p_predict = F * P * F.T + Q
                K = p_predict * H.T / (H * p_predict * H.T + R)
                X = x_predict + K *(Z[0, i] - H * x_predict)
                P = (np.eye(2) - K * H) * p_predict
            lb_list[fed_round] = lb

        new_clients = [clients[i] for i in acc3]
        global_model1 = averageModels1(global_model1, new_clients)
        gl_acc1,gl_loss1 =  test(args, global_model1, device,␣
↪global_test_loader, 'Global', fed_round)

        gl_list[fed_round] = gl_acc
        loss_list[fed_round] = gl_loss
```

```python
        print('\nRound{} set: Average loss for Global model:  Accuracy: ({:.
↪1f}%) Loss: ({:.4f})   Outliers: {}  Filters: {}\n'.
↪format(fed_round+1,gl_acc,loss_list[fed_round],lqd_list[fed_round],lb_list[fed_round]))
        if(fed_round+1 ==args.rounds):
            print(x)
            print(gl_list)
        for  a in range(len(fail_inds)):
            f = fail_inds[a]
            acc_list[fed_round][f] = gl_acc

        # Share the global model with the clients
        for client in clients:
            client['model'].load_state_dict(global_model1.state_dict())

if (args.save_model):

    torch.save(global_model1.state_dict(), "FedAvg.pt")
```

```
Round1 set: Average loss for Global model:  Accuracy: (90.3%) Loss: (1.0756)
Outliers: 14.0  Filters: 0.0


Round2 set: Average loss for Global model:  Accuracy: (93.7%) Loss: (0.5777)
Outliers: 11.0  Filters: 2.0


Round3 set: Average loss for Global model:  Accuracy: (95.5%) Loss: (0.4733)
Outliers: 11.0  Filters: 3.0


Round4 set: Average loss for Global model:  Accuracy: (96.4%) Loss: (0.3715)
Outliers: 9.0  Filters: 2.0


Round5 set: Average loss for Global model:  Accuracy: (96.9%) Loss: (0.3536)
Outliers: 9.0  Filters: 0.0


Round6 set: Average loss for Global model:  Accuracy: (97.2%) Loss: (0.2613)
Outliers: 13.0  Filters: 0.0


Round7 set: Average loss for Global model:  Accuracy: (97.5%) Loss: (0.2426)
Outliers: 9.0  Filters: 0.0
```

```
Round8 set: Average loss for Global model:  Accuracy: (97.7%) Loss: (0.1595)
Outliers: 4.0  Filters: 0.0


Round9 set: Average loss for Global model:  Accuracy: (97.8%) Loss: (0.2036)
Outliers: 4.0  Filters: 1.0


Round10 set: Average loss for Global model:  Accuracy: (97.9%) Loss: (0.1458)
Outliers: 1.0  Filters: 1.0


Round11 set: Average loss for Global model:  Accuracy: (98.1%) Loss: (0.1332)
Outliers: 7.0  Filters: 2.0


Round12 set: Average loss for Global model:  Accuracy: (98.1%) Loss: (0.1483)
Outliers: 4.0  Filters: 1.0


Round13 set: Average loss for Global model:  Accuracy: (98.3%) Loss: (0.0823)
Outliers: 5.0  Filters: 0.0


Round14 set: Average loss for Global model:  Accuracy: (98.3%) Loss: (0.1038)
Outliers: 3.0  Filters: 3.0


Round15 set: Average loss for Global model:  Accuracy: (98.4%) Loss: (0.1735)
Outliers: 3.0  Filters: 2.0


Round16 set: Average loss for Global model:  Accuracy: (98.5%) Loss: (0.2751)
Outliers: 4.0  Filters: 2.0


Round17 set: Average loss for Global model:  Accuracy: (98.4%) Loss: (0.1130)
Outliers: 3.0  Filters: 3.0


Round18 set: Average loss for Global model:  Accuracy: (98.5%) Loss: (0.1137)
Outliers: 5.0  Filters: 4.0


Round19 set: Average loss for Global model:  Accuracy: (98.5%) Loss: (0.1350)
Outliers: 5.0  Filters: 0.0
```

```
Round20 set: Average loss for Global model:  Accuracy: (98.5%) Loss: (0.0910)
Outliers: 5.0  Filters: 1.0


Round21 set: Average loss for Global model:  Accuracy: (98.5%) Loss: (0.2503)
Outliers: 3.0  Filters: 0.0


Round22 set: Average loss for Global model:  Accuracy: (98.5%) Loss: (0.1408)
Outliers: 5.0  Filters: 2.0


Round23 set: Average loss for Global model:  Accuracy: (98.6%) Loss: (0.1033)
Outliers: 6.0  Filters: 3.0


Round24 set: Average loss for Global model:  Accuracy: (98.6%) Loss: (0.0731)
Outliers: 4.0  Filters: 3.0


Round25 set: Average loss for Global model:  Accuracy: (98.6%) Loss: (0.1201)
Outliers: 3.0  Filters: 2.0


Round26 set: Average loss for Global model:  Accuracy: (98.6%) Loss: (0.1010)
Outliers: 5.0  Filters: 2.0


Round27 set: Average loss for Global model:  Accuracy: (98.6%) Loss: (0.0878)
Outliers: 2.0  Filters: 4.0


Round28 set: Average loss for Global model:  Accuracy: (98.7%) Loss: (0.1374)
Outliers: 4.0  Filters: 3.0


Round29 set: Average loss for Global model:  Accuracy: (98.7%) Loss: (0.0900)
Outliers: 4.0  Filters: 1.0


Round30 set: Average loss for Global model:  Accuracy: (98.7%) Loss: (0.0691)
Outliers: 5.0  Filters: 1.0

tensor([222., 262., 270., 246., 210., 220., 218., 210., 250., 200., 270., 230.,
        310., 322., 260., 246., 290., 298., 202., 130., 284., 222., 262., 274.,
        202., 194., 250., 218., 226., 216., 216., 274., 204., 292., 202., 262.,
        210., 306., 258., 194.])
tensor([90.3500, 93.7400, 95.5100, 96.4100, 96.9300, 97.2500, 97.4700, 97.6800,
```

```
        97.7900, 97.9400, 98.0900, 98.0600, 98.2800, 98.2900, 98.3700, 98.4500,
        98.4400, 98.4800, 98.5000, 98.5500, 98.5400, 98.4700, 98.5700, 98.5800,
        98.6200, 98.6100, 98.6300, 98.6600, 98.6800, 98.6600])
```

[14]:
```python
print(loss_list)
```

```
tensor([1.0756, 0.5777, 0.4733, 0.3715, 0.3536, 0.2613, 0.2426, 0.1595, 0.2036,
        0.1458, 0.1332, 0.1483, 0.0823, 0.1038, 0.1735, 0.2751, 0.1130, 0.1137,
        0.1350, 0.0910, 0.2503, 0.1408, 0.1033, 0.0731, 0.1201, 0.1010, 0.0878,
        0.1374, 0.0900, 0.0691])
```

[15]:
```python
print(lqd_list)
```

```
tensor([14., 11., 11.,  9.,  9., 13.,  9.,  4.,  4.,  1.,  7.,  4.,  5.,  3.,
         3.,  4.,  3.,  5.,  5.,  5.,  3.,  5.,  6.,  4.,  3.,  5.,  2.,  4.,
         4.,  5.])
```

[16]:
```python
print(lb_list)
```

```
tensor([0., 2., 3., 2., 0., 0., 0., 0., 1., 1., 2., 1., 0., 3., 2., 2., 3., 4.,
        0., 1., 0., 2., 3., 3., 2., 2., 4., 3., 1., 1.])
```

[17]:
```python
print(acc_list[29])
```

```
tensor([ 98.4000,  99.0000,  99.0000,  98.0000,  98.6600,  98.2857,  87.3333,
         94.0000,  99.6000,  95.0000,  97.7143,  98.5714,  98.8889,  98.8000,
         98.6600,  99.0000,  99.0000,  98.5000,  92.0000,  98.6600,  98.0000,
         98.6600,  99.2000,  98.7500,  98.6600,  98.5000,  96.6667,  75.2500,
         93.6000,  95.0000,  98.6600, 100.0000,  98.6600,  99.2000,  98.6600,
         98.0000,  97.7500, 100.0000,  98.0000,  96.0000])
```
