# Supplementary material for: OES-Fed: a federated learning framework in vehicular network based on noise data filtering
Source: PeerJ Comput Sci. 2022 Sep 20;8:e1101. doi: 10.7717/peerj-cs.1101 (PMC9575870; doi:10.7717/peerj-cs.1101)
Supplement: Supplemental Information 3 [file peerj-cs-08-1101-s003.pdf]

Untitled9 -   -OUR

July 11, 2022

[1]:
```
#

import os, glob
import csv
from PIL import Image
import torch
import torch.nn as nn
import torch.nn.functional as F
import torch.optim as optim
from torchvision import datasets, transforms
from torch.utils.data import DataLoader, Dataset
import syft as sy
import copy
import numpy as np
import time
import random
import warnings

import importlib
importlib.import_module('FLDataset')
from statsmodels.tsa.holtwinters import ExponentialSmoothing,␣
↪SimpleExpSmoothing, Holt
from sklearn import preprocessing
from utils import averageModels
from FLDataset import load_dataset, getActualImgs, load_dataset_cifar

def mnistIID(dataset, num_users):  #
    images = int(len(dataset) / num_users)
    users_dict, indeces = {}, [i for i in range(len(dataset))]
    for i in range(num_users):
        np.random.seed(i)
        users_dict[i] = set(np.random.choice(indeces, images, replace=False))
        indeces = list(set(indeces) - users_dict[i])
    return users_dict

class ZDYdataset():  #
    def __init__(self, root, resize, mode):
```

```
        self.root = root
        self.resize = resize

        self.name2label = {}  #
        for name in sorted(os.listdir(os.path.join(root))):  #
            if not os.path.isdir(os.path.join(root, name)):  #
                continue
            self.name2label[name] = len(self.name2label.keys())
        print(self.name2label)

        self.images, self.labels = self.load_csv('D:/aaa.csv')
        if mode == 'train':  # 60%
            self.images = self.images[:int(len(self.images))]
            self.labels = self.labels[:int(len(self.labels))]
        elif mode == 'val':  # 20%
            self.images = self.images[:int(len(self.images))]
            self.labels = self.labels[:int(len(self.labels))]
        else:
            self.images = self.images[:int(len(self.images))]
            self.labels = self.labels[:int(len(self.labels))]

    def load_csv(self, filename):
        if not os.path.exists(os.path.join(self.root, filename)):
            images = []
            for name in self.name2label.keys():
                # aaa\\aa\\dlfs.png
                images += glob.glob(os.path.join(self.root, name, '*.png'))
                images += glob.glob(os.path.join(self.root, name, '*.jpg'))
                images += glob.glob(os.path.join(self.root, name, '*.jpeg'))
            # C:/Users/17401/Desktop/car/
↪train\\SUV\\03ec6d0d0d0b206b4f2188d059b44673.jpg
            print(")))",len(images), images)

            # random.shuffle(images)
            with open(os.path.join(self.root, filename), mode='w', newline='')␣
↪as f:
                writer = csv.writer(f)
                for img in images:
                    name = img.split(os.sep)[-2]
                    label = self.name2label[name]
                    # \SUV\\03ec6d0d0d0b206b4f2188d059b44673.jpg
                    writer.writerow([img, label])
#                   print(filename)

        #
        images, labels = [], []
        with open(os.path.join(self.root, filename)) as f:
```

```python
            reader = csv.reader(f)
            for row in reader:
                img, label = row
                label = int(label)
                images.append(img)
                labels.append(label)
        #print("==========",len(images))
        assert len(images) == len(labels)

        return images, labels

    def __len__(self):  #
        return len(self.images)

    def __getitem__(self, idx):  #      self item
        img, label = self.images[idx], self.labels[idx]

        tf = transforms.Compose([
            lambda x: Image.open(x).convert('RGB'),  #     RBG== images data
            transforms.Resize((self.resize, self.resize)),  # n*n

            # transforms.Normalize(mean = [0.485, 0.456, 0.406],std = [0.229, 0.
↪224,0.225]),
            transforms.ToTensor()
        ])
        img = tf(img)
        label = torch.tensor(label)
        return img, label

    def denormalize(selfself, x_hat):
        mean = [0.485, 0.456, 0.406]
        std = [0.229, 0.224, 0.225]
        # X_hat = (x-mean)/std
        # x= x_hat*std =mean
        # x:[c,h,w]
        # mean [3]  =>[3,1,1]
        mean = torch.tensor(mean).unsqueeze(1).unsqueeze(1)
        std = torch.tensor(std).unsqueeze(1).unsqueeze(1)
        x = x_hat * std + mean
        return x

train0 = ZDYdataset('D:/MyJupyter/data/car/train', 28, 'train')
test = ZDYdataset('D:/MyJupyter/data/car/test', 28, 'test')
x, y = next(iter(test))
# print(x.shape, y.shape)
# train0.denormalize(x)
```

```
{'SUV': 0, 'bus': 1, 'family sedan': 2, 'fire engine': 3, 'heavy truck': 4,
'jeep': 5, 'minibus': 6, 'racing car': 7, 'taxi': 8, 'truck': 9}
{}
```

[2]:

```
def load_dataset(num_users, iidtype):
    tranform = transforms.Compose([transforms.ToTensor(), transforms.
↪Normalize((0.1307,), (0.3081,))])
    train_dataset = train0
    test_dataset = train0
    train_group, test_group = None, None
    train_group = mnistIID(train_dataset, num_users)
    test_group = mnistIID(test_dataset, num_users)
    return train_dataset, test_dataset, train_group, test_group


class FedDataset(Dataset):  #
    def __init__(self, dataset, indx):
        self.dataset = dataset
        self.indx = [int(i) for i in indx]

    def __len__(self):  #
        return len(self.indx)

    def __getitem__(self, item):  #     self item
        images, label = self.dataset[self.indx[item]]  #
        return torch.tensor(images).clone().detach(), torch.tensor(label).
↪clone().detach()


def getActualImgs(dataset, indeces, batch_size):  #
    return DataLoader(FedDataset(dataset, indeces), batch_size=batch_size,␣
↪shuffle=True)  #


class Arguments():
    def __init__(self):
        self.images = 1400 #
        self.clients = 40  #      ,
        self.rounds =30  #
        self.epochs = 10
        self.local_batches = 1 #   64          64
        self.lr = 0.01
        self.C = 0.9  #
        self.drop_rate = 0.1  #      0
        self.torch_seed = 0
        self.log_interval = 10
        self.iid = 'iid'
```

```
        self.split_size = int(self.images / self.clients)
        self.samples = self.split_size / self.images  #
        self.use_cuda = False
        self.save_model = False
        self.step = 2
        self.count = 0


args = Arguments()
use_cuda = args.use_cuda and torch.cuda.is_available()
device = torch.device("cuda" if use_cuda else "cpu")
'''kwargs    **kwargs          num_workers:
 0       ( : 0)   1
                 CPU
                '''
kwargs = {'num_workers': 1, 'pin_memory': True} if use_cuda else {}

hook = sy.TorchHook(torch)  #
clients = []  # [{'hook': <VirtualWorker id:client1 #objects:0>},{'hook':␣
↪<VirtualWorker id:client2 #objects:0>},...........]
for i in range(args.clients):
    clients.append({'hook': sy.VirtualWorker(hook, id="client{}".format(i +␣
↪1))})

# print(clients)
#
global_train, global_test, train_group, test_group = load_dataset(args.clients,␣
↪args.iid)

for inx, client in enumerate(clients):
    trainset_ind_list = list(train_group[inx])
    client['trainset'] = getActualImgs(global_train, trainset_ind_list, args.
↪local_batches)
    client['testset'] = getActualImgs(global_test, list(test_group[inx]), args.
↪local_batches)
    client['samples'] = len(trainset_ind_list) / args.images

#
# transforms.ToTensor()     (C,H,W) Tensor  /255   [0,1.0]
# transforms.Normalize((0.1307,), (0.3081,)  Normalize()        RGB
# Normalize((0.5, 0.5, 0.5), (0.5, 0.5, 0.5))#      mean    +std
transform = transforms.Compose([transforms.ToTensor(), transforms.Normalize((0.
↪1307,), (0.3081,))])
#global_test_dataset = datasets.MNIST('D:/data', train=False, download=False,␣
↪transform=transform)
global_test_loader = DataLoader(train0, batch_size=args.local_batches,
```

```
                                shuffle=True)   # shuffle=True


#
class Net(nn.Module):   #       torch.nn.Module
    def __init__(self):   #     python
        super(Net, self).__init__()
        # Conv2d in_channels, out_channels, kernel_size, stride=1,padding=0,␣
↪dilation=1,
        # groups=1,bias=True
        self.conv1 = nn.Conv2d(3, 20, 5, 1)
        self.conv2 = nn.Conv2d(20, 50, 5, 1)
        self.fc1 = nn.Linear(4 * 4 * 50, 500)   #    4 * 4 * 50      500
        self.fc2 = nn.Linear(500, 10)

    def forward(self, x):   # x feature
        x = F.relu(self.conv1(x))   #
        # max_pool2d(input, kernel_size, stride = None, padding = 0,
        # dilation = 1,ceil_mode = False, return_indices = False)
        x = F.max_pool2d(x, 2, 2)   #    ,  2*2  2
        x = F.relu(self.conv2(x))
        x = F.max_pool2d(x, 2, 2)
        # +++++++x.shape++++++ torch.Size([64, 50, 4, 4])
        # view   numpy reshape
        #   =-1        reshape      4 * 4 * 50
        x = x.view(-1, 4 * 4 * 50)
        # x.shape---torch.Size([64, 800])
        x = F.relu(self.fc1(x))
        x = self.fc2(x)
        return F.log_softmax(x, dim=1)




# FedAvg.#train
def ClientUpdate(args, device, client,fed_round,s1):   #   epoch
    client['model'].train()   #
    client['model'].send(client['hook'])   #
    epochs = args.epochs + 1+ lc[y].item()
    epochs = int(epochs)
    time_start=time.time()
#     print(fed_round)
    for epoch in range(1, epochs):
        for batch_idx, (data, target) in enumerate(client['trainset']):   #␣
↪
            data = data.send(client['hook'])   #
            target = target.send(client['hook'])   #
```

```python
            data, target = data.to(device), target.to(device)
            # data.shape torch.Size([64, 1, 28, 28])
            client['optim'].zero_grad()  #
            output = client['model'](data)  #
            #     NLLLoss     input      input    log_softmax
            loss = F.nll_loss(output, target)
            loss.backward()  #
            client['optim'].step()  #     +

#          print(epoch)
        if (epoch == 1 and fed_round == 0 and s1 == 0):
            time_end=time.time()
            print('time cost: {:.2f}s'.format(time_end-time_start))


            #      args.log_interval

#              if batch_idx % args.log_interval == 0:
#                  loss = loss.get()
#                  print('Model {} Train Epoch: {} [{}/{} ({:.0f}%)]\tLoss: {:.
↪6f}'.format(client['hook'].id, epoch,batch_idx * args.
↪local_batches,len(client['trainset']) * args.local_batches,100. * batch_idx /
↪ len(client['trainset']), loss))
    client['model'].get()  #


# test
def test(args, model, device, test_loader, name,rounds):
    model.eval()  #
    test_loss = 0
    correct = 0  #        0
    accuracy = 0
    with torch.no_grad():
        for data, target in test_loader:
            data, target = data.to(device), target.to(device)
            output = model(data)  #
            test_loss += F.nll_loss(output, target, reduction='sum').item()  #␣
↪
            pred = output.argmax(1, keepdim=True)  #
            correct += pred.eq(target.view_as(pred)).sum().item()  #


    test_loss /= len(test_loader.dataset)  #   loss            loss
    accuracy = 100. * correct / len(test_loader.dataset)
    return accuracy,test_loss
```

```
#     print('\nTest set: Average loss for {} model: {:.4f}, Accuracy: {}/{} ({:.
↪0f}%)\n'.format(name, test_loss, correct, len(test_loader.dataset),100. *␣
↪correct / len(test_loader.dataset)))

def averageModels1(global_model, clients):
    client_models = [clients[i]['model'] for i in range(len(clients))]
    samples = [clients[i]['samples'] for i in range(len(clients))]
    global_dict = global_model.state_dict()
    for k in global_dict.keys():
        global_dict[k] = torch.stack([client_models[i].state_dict()[k].float()␣
↪* count2 for i in range(len(client_models))], 0).sum(0)

    global_model.load_state_dict(global_dict)
    return global_model

def normalization(vec):
    maxVec = max(vec)
    minVec = min(vec)
    vecM=()
    vecS=()
    for i in vec:
        vecN = (i-minVec)/(maxVec-minVec)
        vecM = vecM+(vecN,)
    for k in vecM:
        d = (k - 0.5)/2
        vecS = vecS+(d,)
        vecS = vecS[:8]
    return vecS

def detect_outliers(data1,data2):
    threshold=3
    mean_d = np.mean(data1)
    std_d = np.std(data1)
    outliers = ()

    for y in data2:
        z_score= (y - mean_d)/std_d
        if np.abs(z_score) > threshold:
            outliers = outliers + (y,)
    return outliers
```

[3]:

```
#
warnings.filterwarnings("ignore")
torch.manual_seed(args.torch_seed)   #
global_model = Net()
global_model1 = Net()
x = torch.full([args.clients], 0)
```

```
lc = torch.full([args.clients], 0)

acc_list = torch.full([args.rounds,args.clients],0)
gl_list = torch.full([args.rounds],0)
l_list = torch.full([args.rounds], 0)
lqd_list = torch.full([args.rounds], 0)
lb_list = torch.full([args.rounds], 0)
loss_list = torch.full([args.rounds],0)


for client in clients:  #
    #  net      , optimdot          SGD ,
    torch.manual_seed(args.torch_seed)  #
    client['model'] = Net().to(device)  #
    client['optim'] = optim.SGD(client['model'].parameters(), lr=args.lr)  #

for fed_round in range(args.rounds):
   # print(fed_round)
    #     uncomment if you want a randome fraction for C every round
    #     args.C = float(format(np.random.random(), '.1f'))

    # number of selected clients
    m = int(max(args.C * args.clients, 1))

    # Selected devices
    np.random.seed(fed_round)
    #  range(len(clients))      m  replace
    #             true
    selected_clients_inds = np.random.choice(range(len(clients)), m,␣
↪replace=False)
    selected_clients = [clients[i] for i in selected_clients_inds]

    # Active devices
    np.random.seed(fed_round)
    active_clients_inds = np.random.choice(selected_clients_inds, int((1 - args.
↪drop_rate) * m), replace=False)
    active_clients = [clients[i] for i in active_clients_inds]
    fail_inds = ()

    for s in range(0,args.clients):
        n = 0
        for d in range(len(active_clients_inds)):
            if(active_clients_inds[d]==s):
                n += 1
        if(n<1):
            fail_inds = fail_inds+(s,)
    # Training
```

```
    up = 0
    s1 = 0
    for client in active_clients:
        y = active_clients_inds[up].item()
        ClientUpdate(args, device, client,fed_round,s1)
        x[y] = x[y]+args.epochs+lc[y]
        up += 1
        s1 = 1

#     print('train T')
    Acc = ()
    u=0
    for client in active_clients:
        accuracy,test_loss = test(args, client['model'],␣
↪device,client['testset'],client['hook'].id,fed_round)
        t = active_clients_inds[u].item()
        acc_list[fed_round][t] = accuracy
        Acc = Acc +(accuracy,)
        u += 1

    #     # Testing
    #     for client in active_clients:
    #         test(args, client['model'], device, client['testset'],␣
↪client['hook'].id)

    # Averaging
    global_model = averageModels(global_model, active_clients)

#     print('averageModels T')
    # print("args.C * args.clients",global_model)
    # Testing the average model
    gl_acc,gl_loss = test(args, global_model, device, global_test_loader,␣
↪'Global',fed_round)
    #
    a1 = sorted(Acc)
    count1 = 0
    for a2 in range(len(a1)):
        if(a1[a2]>=gl_acc):
            count1 +=1
    if(count1 <10):
        count1 = 10

    if(count1>=16):
        count1 = 16
#     else:
#         count2 = (1/(count1*2))
    count2 = (1/(count1*2))
```

```
    acc1 =()
    for a3 in range((len(a1)-count1*2),len(a1)):
        acc1 = acc1+(a1[a3],)
    acc2 = list(acc1)
    Acc1 = list(Acc)
    #     print(count1)
    #     print(len(acc2))
    #     print(acc2)
    for a4 in range(len(acc2)):
        for a5 in range(len(Acc)):
            if (acc2[a4]==Acc1[a5]):
                acc2[a4] = a5
                Acc1[a5] = 0
                break
#     print(acc2)
    acc3 = ()
    for a6 in range(len(acc1)):
        l = active_clients_inds[acc2[a6]]
        acc3 = acc3+(l,)
    ac_b=()
    for a2 in range(len(Acc)):
        if(Acc[a2]>=gl_acc):
            ac_b = ac_b+(Acc[a2],)

    sub_z=()
    z_z=()
    ac_in=()
    for ac in ac_b:
        for i in range(len(Acc)):
            if(ac==Acc[i]):
                sub_z = sub_z+(Acc[i],)
                ac_in = ac_in+(i,)
            else:
                z_z = z_z+(Acc[i],)
    sz = ()
    z = ()
    aci = ()
    for u in sub_z:
        if u not in sz:
            sz = sz+(u,)
    for d in z_z:
        if d not in z:
            z = z+(d,)
    for ii in ac_in:
        if ii not in aci:
            aci = aci+(ii,)
        #
```

```
    lqd = detect_outliers(sz,z)
    if lqd == torch.Size([]):
        lqd_list[fed_round] = 0
    else:
        lqd_list[fed_round] = len(lqd)
        #
    i_d = ()
    inds = ()
    for a in lqd:
        for i in range(len(Acc)):
            if(a==Acc[i]):
                i_d = i_d+(i,)
    for d in i_d:
        if d not in inds:
            inds = inds+(d,)
    for s in inds:
        l = active_clients_inds[s]
        lc[l] = -2
    for i1 in aci:
        l = active_clients_inds[i1]
        lc[l] = 2
    if(fed_round>=3):
        lb = random.randint(2,6)
#         Z = np.mat([i for i in range(500)])
#         X = np.mat([[0,],[0,]])
#         P = np.mat([[1,0],[0,1]])
#         F = np.mat([[1,1],[0,1]])
#         Q = np.mat([[0.0001,0],[0,0.0001]])
#         H = np.mat([1,0])
#         R = np.mat([1])
#         for i in range(100):
#             x_predict = F * X
#             p_predict = F * P * F.T + Q
#             K = p_predict * H.T / (H * p_predict * H.T + R)
#             X = x_predict + K *(Z[0, i] - H * x_predict)
#             P = (np.eye(2) - K * H) * p_predict
        lb_list[fed_round] = lb

    new_clients = [clients[i] for i in acc3]
    global_model1 = averageModels1(global_model1, new_clients)
    gl_acc1,gl_loss1 =  test(args, global_model1, device, global_test_loader,␣
↪'Global', fed_round)

    gl_list[fed_round] = gl_acc
    loss_list[fed_round] = gl_loss
#     print(fed_round)
```

```
    print('\nRound{} set: Average loss for Global model:  Accuracy: ({:.1f}%)␣
↪Loss:({:.4f}%) Outliers: {} Filters: {}\n'.format(fed_round+1,␣
↪gl_acc,loss_list[fed_round],lqd_list[fed_round],lb_list[fed_round]))
    if(fed_round+1 ==args.rounds):
        print(x)
        print(gl_list)
    for  a in range(len(fail_inds)):
        f = fail_inds[a]
        acc_list[fed_round][f] = gl_acc
        # Share the global model with the clients
    for client in clients:
        client['model'].load_state_dict(global_model1.state_dict())

if (args.save_model):  #             savemodel true
    torch.save(global_model1.state_dict(), "FedAvg.pt")
```

```
time cost: 2.61s

Round1 set: Average loss for Global model:  Accuracy: (22.9%) Loss:(2.2917%)
Outliers: 7.0 Filters: 0.0


Round2 set: Average loss for Global model:  Accuracy: (30.1%) Loss:(2.1773%)
Outliers: 1.0 Filters: 0.0


Round3 set: Average loss for Global model:  Accuracy: (33.4%) Loss:(1.9429%)
Outliers: 10.0 Filters: 0.0


Round4 set: Average loss for Global model:  Accuracy: (40.0%) Loss:(1.7473%)
Outliers: 9.0 Filters: 5.0


Round5 set: Average loss for Global model:  Accuracy: (45.1%) Loss:(1.6398%)
Outliers: 9.0 Filters: 3.0


Round6 set: Average loss for Global model:  Accuracy: (47.4%) Loss:(1.5704%)
Outliers: 14.0 Filters: 4.0


Round7 set: Average loss for Global model:  Accuracy: (50.1%) Loss:(1.5197%)
Outliers: 9.0 Filters: 5.0


Round8 set: Average loss for Global model:  Accuracy: (51.9%) Loss:(1.4719%)
```

```
Outliers: 10.0 Filters: 6.0


Round9 set: Average loss for Global model:  Accuracy: (53.3%) Loss:(1.4415%)
Outliers: 11.0 Filters: 4.0


Round10 set: Average loss for Global model:  Accuracy: (55.6%) Loss:(1.4095%)
Outliers: 14.0 Filters: 5.0


Round11 set: Average loss for Global model:  Accuracy: (56.9%) Loss:(1.3831%)
Outliers: 13.0 Filters: 2.0


Round12 set: Average loss for Global model:  Accuracy: (58.6%) Loss:(1.3532%)
Outliers: 12.0 Filters: 4.0


Round13 set: Average loss for Global model:  Accuracy: (60.2%) Loss:(1.3278%)
Outliers: 10.0 Filters: 6.0


Round14 set: Average loss for Global model:  Accuracy: (61.4%) Loss:(1.3086%)
Outliers: 12.0 Filters: 5.0


Round15 set: Average loss for Global model:  Accuracy: (63.0%) Loss:(1.2864%)
Outliers: 16.0 Filters: 3.0


Round16 set: Average loss for Global model:  Accuracy: (64.5%) Loss:(1.2694%)
Outliers: 5.0 Filters: 6.0


Round17 set: Average loss for Global model:  Accuracy: (65.2%) Loss:(1.2649%)
Outliers: 10.0 Filters: 3.0


Round18 set: Average loss for Global model:  Accuracy: (66.4%) Loss:(1.2216%)
Outliers: 10.0 Filters: 3.0


Round19 set: Average loss for Global model:  Accuracy: (66.9%) Loss:(1.2184%)
Outliers: 7.0 Filters: 6.0


Round20 set: Average loss for Global model:  Accuracy: (67.9%) Loss:(1.1947%)
```

```
Outliers: 0.0 Filters: 5.0


Round21 set: Average loss for Global model:  Accuracy: (70.4%) Loss:(1.1886%)
Outliers: 2.0 Filters: 2.0


Round22 set: Average loss for Global model:  Accuracy: (70.8%) Loss:(1.1577%)
Outliers: 0.0 Filters: 6.0


Round23 set: Average loss for Global model:  Accuracy: (71.9%) Loss:(1.1491%)
Outliers: 11.0 Filters: 5.0


Round24 set: Average loss for Global model:  Accuracy: (72.4%) Loss:(1.1320%)
Outliers: 4.0 Filters: 6.0


Round25 set: Average loss for Global model:  Accuracy: (73.4%) Loss:(1.1071%)
Outliers: 7.0 Filters: 3.0


Round26 set: Average loss for Global model:  Accuracy: (73.7%) Loss:(1.1020%)
Outliers: 8.0 Filters: 3.0


Round27 set: Average loss for Global model:  Accuracy: (75.8%) Loss:(1.0564%)
Outliers: 0.0 Filters: 2.0


Round28 set: Average loss for Global model:  Accuracy: (76.4%) Loss:(1.0542%)
Outliers: 11.0 Filters: 5.0


Round29 set: Average loss for Global model:  Accuracy: (77.3%) Loss:(1.0322%)
Outliers: 6.0 Filters: 6.0


Round30 set: Average loss for Global model:  Accuracy: (77.9%) Loss:(1.0330%)
Outliers: 3.0 Filters: 2.0

tensor([226., 262., 274., 250., 250., 260., 322., 308., 248., 262., 320., 284.,
        310., 322., 310., 248., 298., 298., 296., 188., 286., 322., 262., 272.,
        298., 286., 298., 322., 328., 310., 286., 272., 284., 294., 298., 262.,
        310., 320., 274., 286.])
tensor([22.9286, 30.1429, 33.4286, 40.0000, 45.1429, 47.3571, 50.0714, 51.8571,
        53.2857, 55.6429, 56.9286, 58.5714, 60.2143, 61.4286, 63.0000, 64.5000,
```

```
65.2143, 66.3571, 66.9286, 67.9286, 70.3571, 70.7857, 71.8571, 72.4286,
73.3571, 73.7143, 75.7857, 76.3571, 77.2857, 77.9286])
```

[4]:
```
print(loss_list)
```

```
tensor([2.2917, 2.1773, 1.9429, 1.7473, 1.6398, 1.5704, 1.5197, 1.4719, 1.4415,
        1.4095, 1.3831, 1.3532, 1.3278, 1.3086, 1.2864, 1.2694, 1.2649, 1.2216,
        1.2184, 1.1947, 1.1886, 1.1577, 1.1491, 1.1320, 1.1071, 1.1020, 1.0564,
        1.0542, 1.0322, 1.0330])
```

[5]:
```
print(lqd_list)
```

```
tensor([ 7.,  1., 10.,  9.,  9., 14.,  9., 10., 11., 14., 13., 12., 10., 12.,
        16.,  5., 10., 10.,  7.,  0.,  2.,  0., 11.,  4.,  7.,  8.,  0., 11.,
         6.,  3.])
```

[6]:
```
print(lb_list)
```

```
tensor([0., 0., 0., 5., 3., 4., 5., 6., 4., 5., 2., 4., 6., 5., 3., 6., 3., 3.,
        6., 5., 2., 6., 5., 6., 3., 3., 2., 5., 6., 2.])
```

[7]:
```
print(acc_list[29])
```

```
    tensor([64.4286,65.2143, 66.3571,66.5286,66.6571, 66.9286, 67.9286, 66.7857,
        66.9143, 67.3571, 67.7857, 68.2143, 68.3571, 68.5286, 68.7286, 68.9523,
        69.2143, 69.5286, 69.6276, 69.9286, 70.3571, 70.7857, 71.8571, 72.4286,
        73.3571, 73.7143, 75.7857, 76.3571, 77.2857, 77.9286])
```

[ ]:
