# Supplementary material for: OES-Fed: a federated learning framework in vehicular network based on noise data filtering
Source: PeerJ Comput Sci. 2022 Sep 20;8:e1101. doi: 10.7717/peerj-cs.1101 (PMC9575870; doi:10.7717/peerj-cs.1101)
Supplement: Supplemental Information 4 [file peerj-cs-08-1101-s004.pdf]

In [1]:
```
import matplotlib.pyplot as plt
import seaborn as sns
import numpy as np
```

In [2]:
```
x = [1,2,3,4,5,6,7,8,9,10,11,12,13,14,15,16,17,18,19,20,21,22,23,24,25,26,27,28,29,30]
lqd_c = [32., 35., 29., 30., 30., 25., 30., 29., 28., 25., 26., 27., 29., 27.,23., 34.
lb_c = [32, 35, 29, 27., 28., 20., 27., 24., 22., 22., 22., 21., 23., 22., 18., 31., 2
lqd_m = [26., 29., 29., 31., 31., 27., 31., 36., 36., 39., 33., 36., 35., 37.,37., 36.
lb_m = [26., 29, 29, 29.,31., 27., 31., 36., 35., 38., 31., 35., 35., 34., 35., 34., 3
```

In [3]:
```
c1 = []
c2 = []
for i in lb_m:
    c1.append(i-8)
for i in lqd_m:
    c2.append(i-8)
```

In [4]:
```
c3 = []
c4 = []
for i in lb_c:
    c3.append(i-8)
for i in lqd_c:
    c4.append(i-8)
```

In [14]:
```
plt.subplot(1,2,1)
plt.axhline(y=32,ls=":",c="black")
plt.plot(x, c2,  color='black', label='Current Perspective \n of Nosie Data_MNIST',ls
plt.plot(x, c1,  color='red', label='Historical Perspective \n of Noise Data_MNIST',l
plt.fill_between(x, c1, color='red', alpha=0.7)
plt.xlabel('rounds')
plt.ylabel('clients')
plt.legend(prop = {'size':9.5})

plt.subplot(1,2,2)
plt.axhline(y=32,ls=":",c="black")
plt.plot(x, c4,  color='black', label='Current Perspective \n of Nosie Data_CIFAR',ls
plt.plot(x, c3,  color='red', label='Historical Perspective \n of Noise Data_CIFAR',l
plt.fill_between(x, c3, color='red', alpha=0.7)
plt.xlabel('rounds')
plt.legend(prop = {'size':9.5})

plt.savefig("clients.pdf")
```

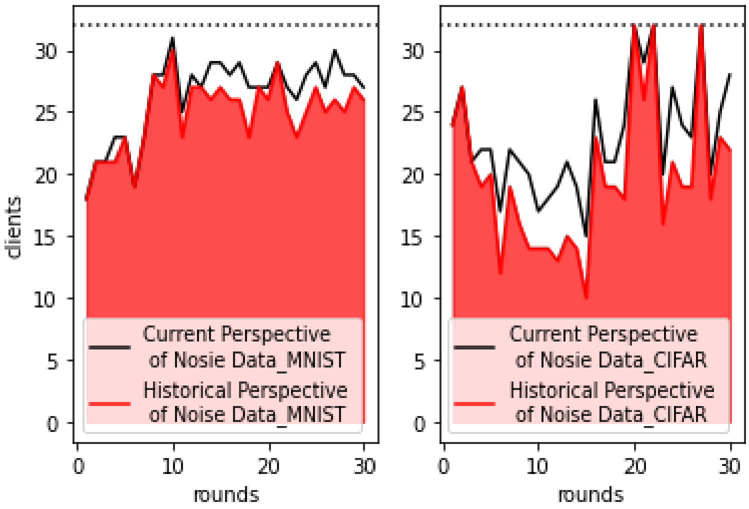

In [6]:
```
acc_avg = [90.7300, 93.4800, 93.9600, 95.3300, 95.6500, 95.5500, 96.2100, 95.9500,96.(
acc_sgd = [10.2300, 10.4700, 10.9200, 11.5800, 12.4400, 13.4300, 14.7800,16.3200, 17.9
```

```
acc_our = [90.3500, 93.7400, 95.5100, 96.4100, 96.9300, 97.2500, 97.4700, 97.6800,97.7

acc_cavg = [21.7500, 21.1200, 20.4700, 20.3100, 20.5300, 20.2400, 20.5400, 20.1900,20.
acc_csgd = [11.7000, 11.7800, 11.8600, 12.0400, 12.1600, 12.2100, 12.2900,12.3800, 12.
acc_cour = [21.7000, 29.3100, 33.6300, 36.6700, 40.2200, 42.5800, 43.5300, 45.8200,46.

loss_avg = [0.8014, 0.6091, 0.3872, 0.4697, 0.3499, 0.4341, 0.5339, 0.5922, 0.5284,0.5
loss_sgd = [2.3083, 2.3038, 2.2993, 2.2949, 2.2906, 2.2863, 2.2821, 2.2779,2.2737, 2.2
loss_our = [1.0756, 0.5777, 0.4733, 0.3715, 0.3536, 0.2613, 0.2426, 0.1595, 0.2036,0.1

loss_cavg = [2.2725, 2.2638, 2.2599, 2.2555, 2.2507, 2.2466, 2.2442, 2.2432, 2.2424,2.
loss_csgd = [2.3022, 2.3020, 2.3018, 2.3015, 2.3013, 2.3011, 2.3009, 2.3006,2.3004, 2.
loss_cour = [2.2724, 2.2161, 2.1783, 2.1476, 2.1239, 2.1037, 2.0891, 2.0763, 2.0648,2.
```

In [3]:

```
61.7500-19.7300
```

Out[3]:

```
42.019999999999996
```

In [20…

```
print(np.mean(acc_avg),np.mean(acc_sgd),np.mean(acc_our))
```

```
95.90066666666667 28.597333333333333 97.68666666666665
```

In [20…

```
print(np.mean(acc_cavg),np.mean(acc_csgd),np.mean(acc_cour))
```

```
20.216666666666665 13.051666666666668 50.299666666666674
```

In [20…

```
print(np.mean(loss_avg),np.mean(loss_sgd),np.mean(loss_our))
```

```
0.4388666666666667 2.2454733333333334 0.21354333333333334
```

In [20…

```
print(np.mean(loss_cavg),np.mean(loss_csgd),np.mean(loss_cour))
```

```
2.2443533333333336 2.2989200000000003 2.005581666666667
```

In [15…

```
f, (ax, ax2) = plt.subplots(2, 1, sharex=True)
ax.set_ylim(90, 100)  # outliers only
ax2.set_ylim(10, 60)
ax.plot(acc_our,  color='red', marker='D',label='OES-Fed in MNIST',ls='-')
ax.plot(acc_avg,  color='black',marker='x', label='FedAVG in MNIST',ls='-')
ax2.plot(acc_sgd,  color='purple',marker='x', label='FedSGD in MNIST',ls='-')
ax.spines['bottom'].set_visible(False)
ax2.spines['top'].set_visible(False)
plt.ylabel('accuracy')
plt.xlabel('rounds')
ax.legend()
ax2.legend(loc=4)
plt.savefig("accm.pdf")
```

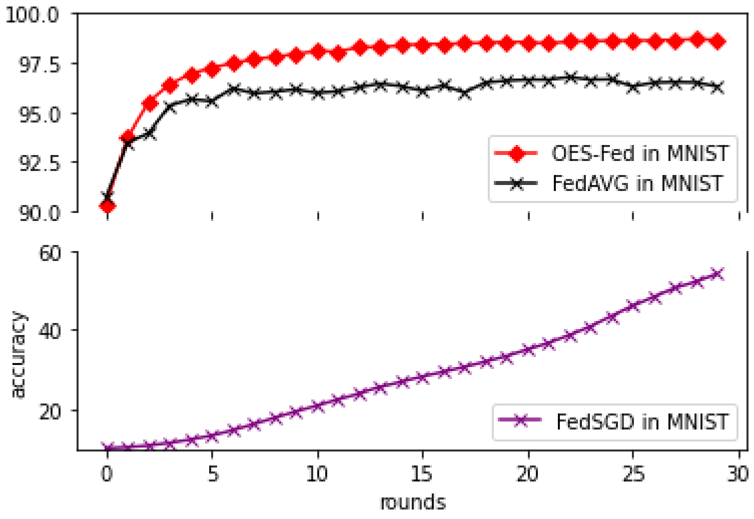

In [16…
```
plt.plot(x, acc_cour,  color='red',marker='D', label='OES-Fed in CIFAR',ls='-')
plt.plot(x, acc_cavg,  color='black',marker='x', label='FedAVG in CIFAR',ls='-')
plt.plot(x, acc_csgd,  color='purple', marker='x',label='FedSGD in CIFAR',ls='-')
plt.xlabel('rounds')
plt.legend(loc=7)
plt.savefig("acc.pdf")
```

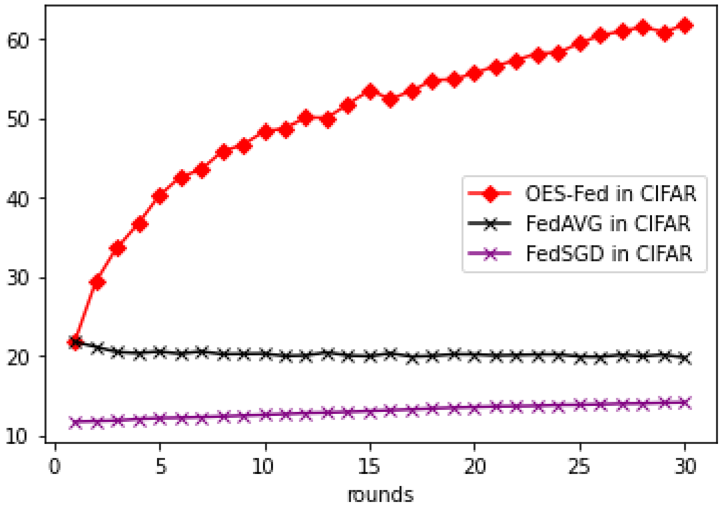

In [16…
```
plt.plot(x, loss_our,  color='red',marker='D', label='OES-Fed in MNIST',ls='-')
plt.plot(x, loss_avg,  color='black',marker='x', label='FedAVG in MNIST',ls='-')
plt.plot(x, loss_sgd,  color='purple',marker='x', label='FedSGD in MNIST',ls='-')
plt.ylabel('loss')
plt.xlabel('rounds')
plt.legend(loc=7)
plt.savefig("lossm.pdf")
```

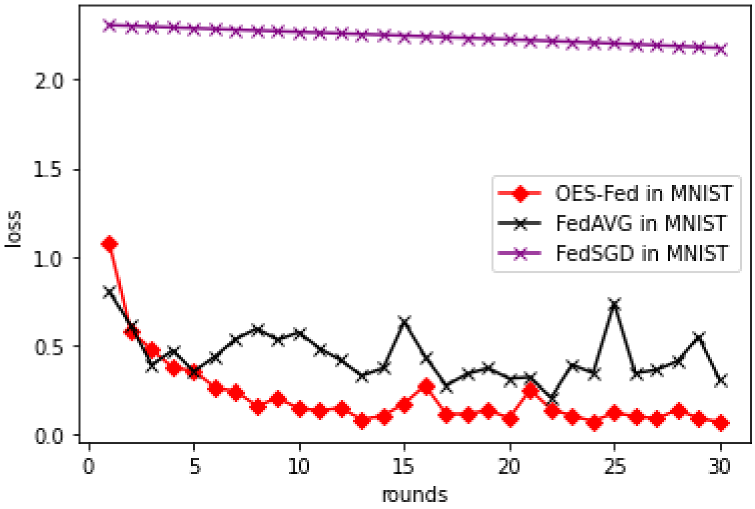

In [16…
```
plt.plot(x, loss_cour,  color='red',marker='D', label='OES-Fed in CIFAR',ls='-')
plt.plot(x, loss_cavg,  color='black',marker='x', label='FedAVG in CIFAR',ls='-')
plt.plot(x, loss_csgd,  color='purple',marker='x', label='FedSGD in CIFAR',ls='-')
plt.ylabel('loss')
plt.xlabel('rounds')
plt.legend(loc=7)
plt.savefig("loss.pdf")
```

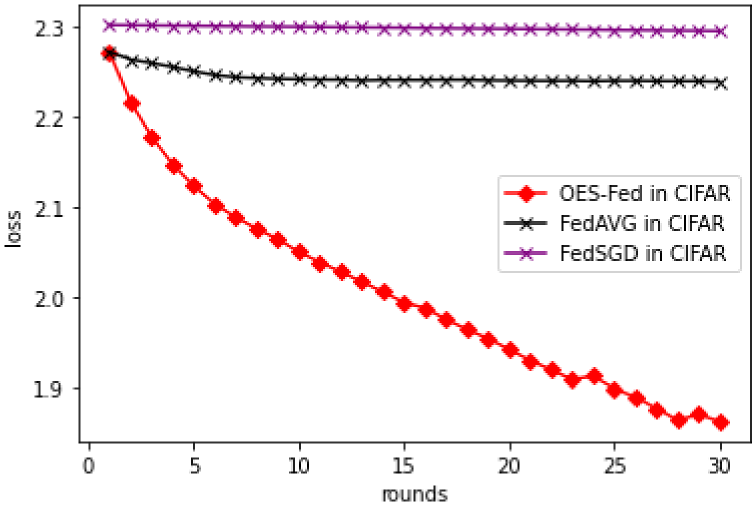

In [18]:
```
num = [1,2,3,4,5,6,7,8,9,10,11,12,13,14,15,16,17,18,19,20,21,22,23,24,25,26,27,28,29,3
lc_c = [186, 230, 190, 196, 184, 210, 278, 234, 202, 178, 218, 230,258, 246, 266, 198,
lc_m = [222, 262, 270, 246, 210, 220, 218, 210, 250, 200, 270, 230,310, 322, 260, 246,
c = sorted(lc_c)
m = sorted(lc_m)
```

In [62]:
```
print(sum(lc_c),sum(lc_m))
```

9092 9632

In [63]:
```
print(np.mean(lc_c),np.mean(lc_m))
```

227.3 240.8

In [19…
```
oum = [98.66,96,98.5,95,92,98.66,98.66,98.66,94,97.75,98.66,95,98.66,75.25,87.3333,98.
fem = [97.2000, 47.5000, 70.5000, 94.5000, 96.3000, 94.0000, 12.8889, 97.3333,98.8000,
om = sorted(oum)
fm = sorted(fem)

ouc = [59.2000, 63.2000, 62.0000, 59.6000, 61.7500, 58.4000, 63.2000, 57.2000,63.6000,
fec = [20.0000, 19.2000, 16.0000, 18.8000,  19.7300, 19.2000, 24.0000, 14.4000,24.800(
oc = sorted(ouc)
fc = sorted(fec)
```

In [20…
```
plt.subplot(1,2,1)
plt.plot(num, om,  color='red', label='OES-Fed in MNIST',ls='-')
plt.plot(num, fm,  color='black', label='FedAVG in MNIST',ls='-')
plt.fill_between(num,fm, om, color='gray',alpha=0.5)
plt.xlabel('Vehicles')
plt.ylabel('Accuracy')
plt.legend()

plt.subplot(1,2,2)
plt.ylim(0,100)
plt.plot(num, oc,  color='red', label='OES-Fed in CIFAR',ls='-')
plt.plot(num, fc,  color='black', label='FedAVG in CIFAR',ls='-')
plt.fill_between(num,fc, oc, color='gray',alpha=0.5)
plt.xlabel('Vehicles')
plt.legend()

plt.savefig("accf.pdf")
```

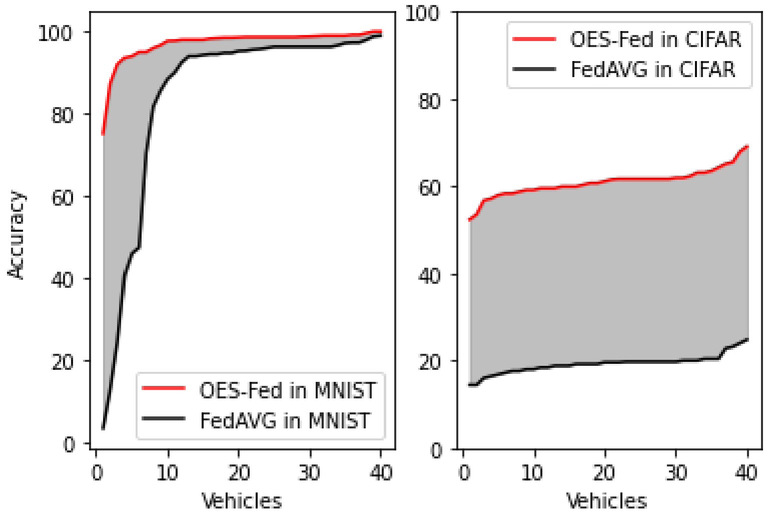

In [ ]:
```
plt.bar(num, c, label='graph 1')
plt.plot(num, a,  color='red', label='CIFAR',ls='-')
plt.xlabel('clients')
plt.ylabel('rounds')
```

In [33]:
```
from mpl_toolkits.axes_grid1 import host_subplot
import mpl_toolkits.axisartist as AA
```

In [30]:
```
acc = [98.66,96,98.5,95,92,98.66,98.66,98.66,94,97.75,98.66,95,98.66,75.25,87.3333,98.
```

In [11…
```
plt.figure(dpi=250,figsize=(35,8))
host = host_subplot(111, axes_class=AA.Axes)
plt.subplots_adjust(right=0.75)
par2 = host.twinx()
offset = 0
new_fixed_axis = par2.get_grid_helper().new_fixed_axis
par2.axis["right"] = new_fixed_axis(loc="right",
         axes=par2,
         offset=(offset, 0))
par2.axis["right"].toggle(all=True)
host.set_xlim(1, 40)
host.set_ylim(0, 500)
p3, = par2.plot(num, acc, label="Accuracy for each vehicle in MNIST",color='red',mark
par2.set_ylim(50, 100)
par2.axis["right"].label.set_color(p3.get_color())
plt.bar(num, m, label='Total rounds for each vehicle in MNIST')
plt.axhline(y=240,ls=":",c="black")
plt.xlabel('Vehicle number')
plt.ylabel('Total rounds')
par2.set_ylabel('Accuracy')

plt.legend(fontsize='xx-large',bbox_to_anchor=(1.05, 0), loc=3, borderaxespad=0)

plt.savefig("lc.pdf")
```

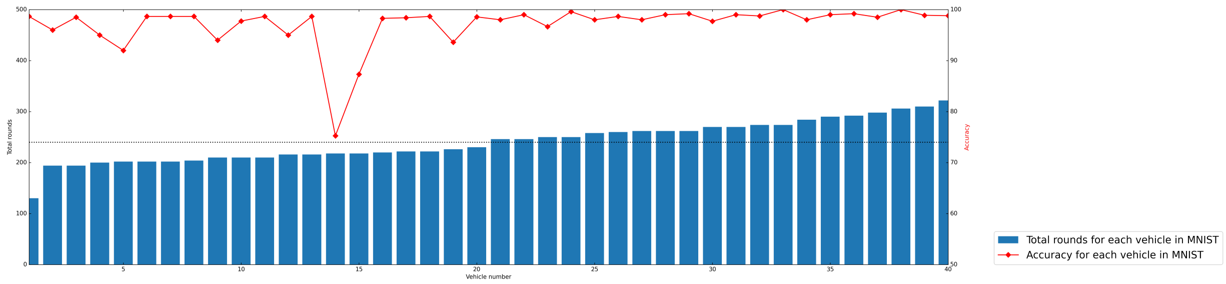

In [10…
```
ac_c = [61.75,58.8,61.75,59.2,62,56.8,59.6,58.4,63.6,60,61.2,53.6,58.4,65.6,60.8,59.2,
```

In [11⋯

```
plt.figure(dpi=250,figsize=(35,8))
host = host_subplot(111, axes_class=AA.Axes)
plt.subplots_adjust(right=0.75)
par2 = host.twinx()
offset = 0
new_fixed_axis = par2.get_grid_helper().new_fixed_axis
par2.axis["right"] = new_fixed_axis(loc="right",
         axes=par2,
         offset=(offset, 0))
par2.axis["right"].toggle(all=True)
host.set_xlim(1, 40)
host.set_ylim(0, 500)
p3, = par2.plot(num, ac_c, label="Accuracy for each vehicle in CIFAR",color='red',mar
par2.set_ylim(30, 100)
par2.axis["right"].label.set_color(p3.get_color())
plt.bar(num, c, label='Total rounds for each vehicle in CIFAR')
plt.axhline(y=240,ls=":",c="black")
plt.xlabel('Vehicle number')
plt.ylabel('Total rounds')
par2.set_ylabel('Accuracy')

plt.legend(fontsize='xx-large',bbox_to_anchor=(1.05, 0), loc=3, borderaxespad=0)

plt.savefig("lcc.pdf")
```

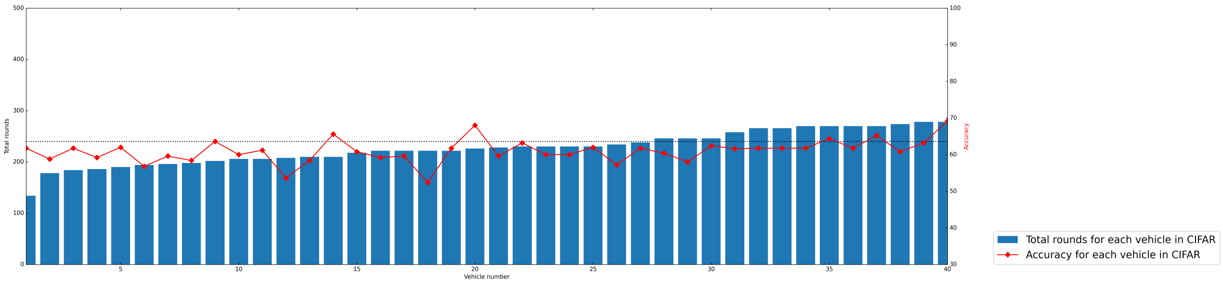

In [2]:

```
from sklearn import metrics
import matplotlib.pyplot as plt
```

In [3]:

```
a1 = ([0.        , 0.14212329, 0.19520548, 0.34075342, 0.40582192,0.52568493, 0.65582
a2 = ([0.        , 0.04807692, 0.16826923, 0.23317308, 0.35336538,0.40144231, 0.47355
b1 = ([0.        , 0.06666667, 0.86453293, 0.86453293, 0.93333333,1.        ])
b2 = ([0.        , 0.03807692, 0.64057692, 0.64057692, 0.67836538,1.        ])
c1 = ([0.        , 0.07692308, 0.68461538, 0.84615385, 0.95615385,1.        ])
c2 = ([0.        , 0.02574386, 0.15469873, 0.25432891, 0.35582192,1.        ])
```

In [4]:

```
auc1 = metrics.auc(a2, a1)
auc2 = metrics.auc(b2, b1)
auc3 = metrics.auc(c2, c1)
```

In [5]:

```
d1 = ([0.   , 0.109, 0.213, 0.31 , 0.413, 0.515, 0.621, 0.726, 0.824,0.907, 1.    ])
d2 = ([0.        , 0.04807692, 0.16826923, 0.23317308, 0.35336538,0.40144231, 0.47355
e1 = ([0.        , 0.06666667, 0.13333333, 0.33333333, 0.33333333,0.46666667, 0.73333
e2 = ([0.        , 0.16826923, 0.23547318, 0.25438736, 0.31257489,0.33294875, 0.48956
f1 = ([0.        , 0.23076923, 0.38461538, 0.69230769, 0.76923077,0.79615385, 0.88472
f2 = ([0.        , 0.16826923, 0.23317308, 0.31467528, 0.33492785,0.33627589, 0.36258
```

In [6]:

```
auc4 = metrics.auc(d2, d1)
auc5 = metrics.auc(e2, e1)
auc6 = metrics.auc(f2, f1)
```

In [9]:

```
plt.figure()
lw = 2
```

```
plt.plot(a1, a2, color='purple',marker='x',lw=lw, label='FedSGD auc = %0.2f' % auc1)
plt.plot(b1, b2, color='black',marker='x',lw=lw, label='FedAVG auc = %0.2f' % auc2)
plt.plot(c1, c2, color='red',marker='D',lw=lw, label='OES-Fed auc = %0.2f' % auc3)
plt.plot([0, 1], [0, 1], color='navy', lw=lw, linestyle='--')
plt.xlim([0.0, 1.0])
plt.ylim([0.0, 1.05])
plt.xlabel('False Positive Rate')
plt.ylabel('True Positive Rate')
plt.title('Receiver operating characteristic example(MNIST)')
plt.legend(loc="upper left")

plt.savefig("11.pdf")
plt.show()
```

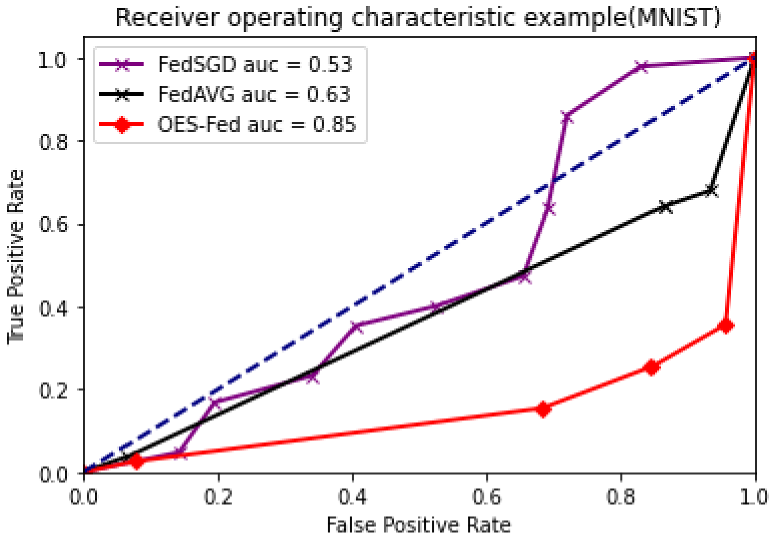

In [12]:

```
plt.figure()
lw = 2

plt.plot(d1, d2, color='purple',marker='x',lw=lw, label='FedSGD auc = %0.2f' % auc4)
plt.plot(e1, e2, color='black',marker='x',lw=lw, label='FedAVG auc = %0.2f' % auc5)
plt.plot(f1, f2, color='red',marker='D',lw=lw, label='OES-Fed auc = %0.2f' % auc6)
plt.plot([0, 1], [0, 1], color='navy', lw=lw, linestyle='--')
plt.xlim([0.0, 1.0])
plt.ylim([0.0, 1.05])
plt.xlabel('False Positive Rate')
plt.ylabel('True Positive Rate')
plt.title('Receiver operating characteristic example(CIFAR-10)')
plt.legend(loc="upper left")

plt.savefig("22.pdf")
plt.show()
```

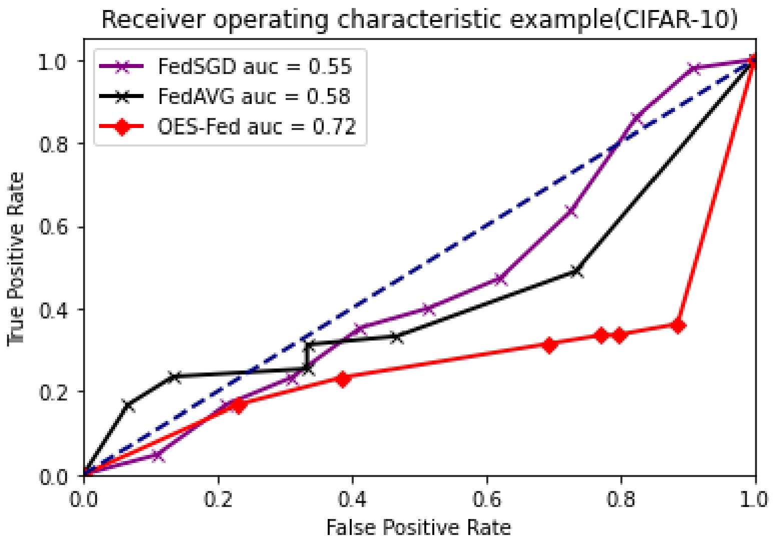
Receiver operating characteristic example(CIFAR-10)
FedSGD auc = 0.55
FedAVG auc = 0.58
OES-Fed auc = 0.72
True Positive Rate
False Positive Rate
0.0
0.2
0.4
0.6
0.8
1.0

In [ ]:
